# Prognostic role of the long non-coding RNA, *SPRY4 Intronic Transcript 1*, in patients with cancer: a meta-analysis

**DOI:** 10.18632/oncotarget.16735

**Published:** 2017-03-31

**Authors:** Miaojuan Wang, Xuejun Dong, Yi Feng, Honggang Sun, Ningping Shan, Tao Lu

**Affiliations:** ^1^ Clinical Laboratory Center of Shaoxing People's Hospital, Shaoxing Hospital of Zhejiang University, Shaoxing, China

**Keywords:** lncRNA, SPRY4-IT1, prognosis, overall survival, meta-analysis

## Abstract

Recent studies have emphasized the important role of long non-coding RNAs (lncRNAs) in cancer development. The present study performed a meta-analysis to investigate whether lncRNA, *SPRY4 Intronic Transcript 1(SPRY4-IT1*) can be served as a potential biomarker for prognosis in human cancers. The eligible studies were collected by searching multiple online databases (Pubmed, EMBASE, CNKI, Web of Science and Google Scholar) and meta-analysis was performed to explore the association between the expression levels of *SPRY4-IT1* and overall survival (OS), disease-free survival (DFS) and clinicopathological parameters. A total of 1329 patients from 13 studies were included for meta-analysis. The meta-analysis results showed that high expression level of *SPRY4-IT1* was significantly associated with shorter OS in cancer patients (HR = 3.20, 95% CI: 2.59-3.90, P<0.001) except in the patients with non-small cell lung cancer (NSCLC). Increased *SPRY4-IT1* expression level was correlated with shorter DFS in patients with gastric cancer and ovarian cancer. *SPRY4-IT1* expression level was not correlated with the clinicopathological parameters including age (P = 0.37), gender (P = 0.87), tumor size (P = 0.47) and invasion depth (P = 0.52), and increased *SPRY4-IT1* expression level was significantly associated with distant metastasis (odds ratio (OR) = 1.96, 95% CI: 1.24-3.08, P = 0.004), lymph node metastasis (OR = 3.96, 95% CI: 1.48-5.54, P<0.001), advanced tumor/node/metastasis stage (OR = 3.72, 95% CI = 2.91-4.76, P<0.001) and poor tumor differentiation (OR = 1.86, 95% CI = 1.35-2.58, P<0.001) in cancer patients except in patients with NSCLC. In summary, the meta-analysis results suggested that increased expression level of *SPRY4-IT1* was positively associated with unfavorable prognosis and advanced features of cancers in cancer patients but not in patients with NSCLC.

## INTRODUCTION

Cancer has become a serious worldwide public health issue, and there are about 14 million new cases of cancer occurred globally, which caused about 8 million of human deaths in 2012 worldwide [1]. Though the surgical techniques and chemotherapy/radiotherapy regimens are with great improvement, the 5-year survival rates of the patients with certain types of cancers are still very low [1, 2]. Because of the insufficient knowledge about molecular mechanisms underlying cancer development, the overall cancer-related deaths were expected to rise in the future. Therefore, identifying novel biomarkers for early diagnosis and prognosis is necessary for us to have a better control of cancer.

The long non-coding RNAs (lncRNAs) are transcribed RNA with more than 200 nt and are incapable of coding proteins [3]. LncRNAs have drawn great attention in various studies because of their diverse cellular functions such cell differentiation, cell proliferation, cell apoptosis and cell survival [4, 5]. Recently, the role of lncRNAs in cancer development has been revealed in numerous studies. For example, the lncRNA, *HOX transcript antisense RNA (HOTAIR)* up-regulation serves as a novel predictive factor for poor prognosis in different types of cancers in both Asian and Western countries [6]. The high expression pattern and oncogenic role of the lncRNA, *colon cancer associated transcript 1 (CCAT1)* was identified in different types of cancer, and the aberrant expression of *CCAT1* is involved in several processes correlated with carcinogenesis such as cell proliferation, apoptosis, migration and invasion by regulating different target genes and pathways [7]. The lncRNA, *HOXA transcript at the distal tip (HOTTIP)* has been widely reported for its role in the initiation and progression of human cancers including hepatocellular carcinoma, pancreatic cancer, gastric cancer and colorectal cancer [8]. The lncRNA, *urothelial cancer-associated 1 (UCA1)* was identified as a common molecular marker for lymph node metastasis and prognosis in various cancers [9]. The lncRNA *SPRY4 intronic transcript 1*(*SPRY4-IT1*) was recently identified in melanoma, and increased expression of *SPRY4-IT1* was closely associated with tumor site and tumor stage, which indicated the prognostic role of *SPRY4-IT1* in patients with melanoma [10, 11]. In addition, the roles of *SPRY4-IT1* in cancer development were also identified in other types of cancers such as cervical cancer, colorectal cancer, lung cancer, breast cancer, liver cancer and so on, and *SPRY4-IT1* was found to be a prognostic factor in these cancers [12–16]. However, the underlying molecular mechanisms in cancer progression are rarely explored.

In the present study, we for the first time performed the meta-analysis to examine the association between the *SPRY4-IT1* expression level and prognosis in cancer patients. In the meta-analysis, eligible studies were included for analysis to examine the potential prognostic role of *SPRY4-IT1* in cancer patients.

## RESULTS

### Eligible studies

**Figure 1:**
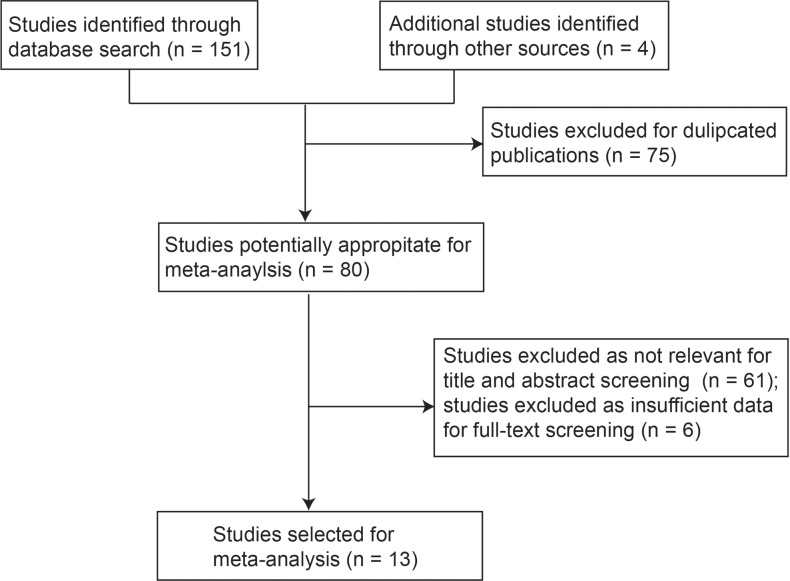
Procedures of selecting eligible studies for meta-analysis

A total of 155 articles were identified by searching different databases. After excluding 75 duplicate public-ations, 80 articles were included for further screening. After carefully reviewing the title and abstract, as well as the full text, 13 studies were finally selected based on the inclusion and exclusion criteria described in the methodology section (Figure 1).

### Study characteristics

A total of 1329 cases from 13 included eligible studies with relevant clinical data were included in this meta-analysis. The year of publication ranges from 2014-2017. All of these studies were conducted in China, and there are 11 types of cancers among the 13 included studies. The lncRNA, *SPRY4-IT1* expression levels in these studies were all measured by quantitative real time PCR (qRT-PCR). Table 1 shows the summary of the main characteristics of the 13 included eligible studies.

**Table 1:** Summary of included eligible studies for meta-analysis in the present study

First Author	Year	Cancer type	Blood or tissue	Total number	Tumor stage	Year of survival	Adjuvant therapy before surgery	Criterion of high expression	Detection method	Outcome measures	Multivariate analysis
Cao D. [12]	2015	Colorectal cancer	Tissue	84	41/43 (I-II/III-IV)	3	None	Cut-off value	qRT-PCR	OS	Yes
Cao Y. [13]	2016	Cervical cancer	Tissue	110	55/45 (I-II/III-IV)	5	None	Youden'x index	qRT-PCR	OS	Yes
Li H. [33]	2017	Ovarian cancer	Tissue	124	48/76 (I-II/III-IV)	5	None	Median expression	qRT-PCR	OS, DFS	Yes
Liu D. [34]	2017	Bladder cancer	Tissue	60	15/45 (I-II/III-IV)	NR	NR	NR	qRT-PCR	NR	NR
Liu T. [11]	2016	Melanoma	Plasma	70	32/38 (I-II/III-IV)	5	None	Cut-off value	qRT-PCR	OS	Yes
Peng W. [24]	2015	Gastric cancer	Tissue	175	95/80 (I-II/III-IV)	5	NR	Median expression	qRT-PCR	OS, DFS	Yes
Shi Y. [15]	2015	Breast cancer	Tissue	48	23/25 (I-II/III-IV)	NR	None	NR	qRT-PCR	NR	NR
Sun M. [16]	2014	NSCLC	Tissue	121	43/78 (I-II/III-IV)	3	None	Median expression	qRT-PCR	OS, DFS	Yes
Tan W. [35]	2017	Colorectal cancer	Tissue	116	57/49 (I-II/III-IV)	5	None	Mean expression	qRT-PCR	OS	Yes
Xie H. [23]	2014	ESCC	Tissue	92	59/33 (I-II/III-IV)	5	None	Median expression	qRT-PCR	OS	Yes
Zhang H. [36]	2014	RCC	Tissue	98	63/35 (I-II/III-IV)	5	None	Mean expression	qRT-PCR	OS	Yes
Zhao X. [22]	2015	Bladder cancer	Tissue	68	32/36(I-II/III-IV)	5	None	Mean expression	qRT-PCR	OS	Yes
Zhou Y. [37]	2016	Glioma	Tissue	163	73/90 (I-II/III-IV)	5	NR	Median expression	qRT-PCR	OS	Yes

DFS = disease-free survival; ESCC = Esophageal squamous cell carcinoma; NR = not recorded; NSCLC = non-small cell lung cancer; OS = overall survival; RCC = renal cell carcinoma.

### Meta-analysis of the association between *SPRY4-IT1* expression level and overall survival (OS)

**Figure 2:**
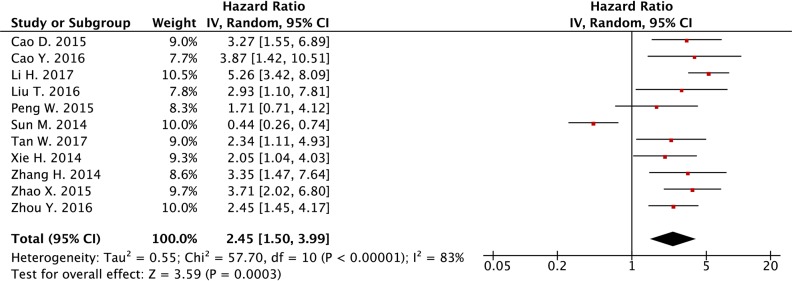
Forest plot of the association between lncRNA *SPRY4-IT1* expression level and overall survival in cancer patients from 11 studies

**Figure 3:**
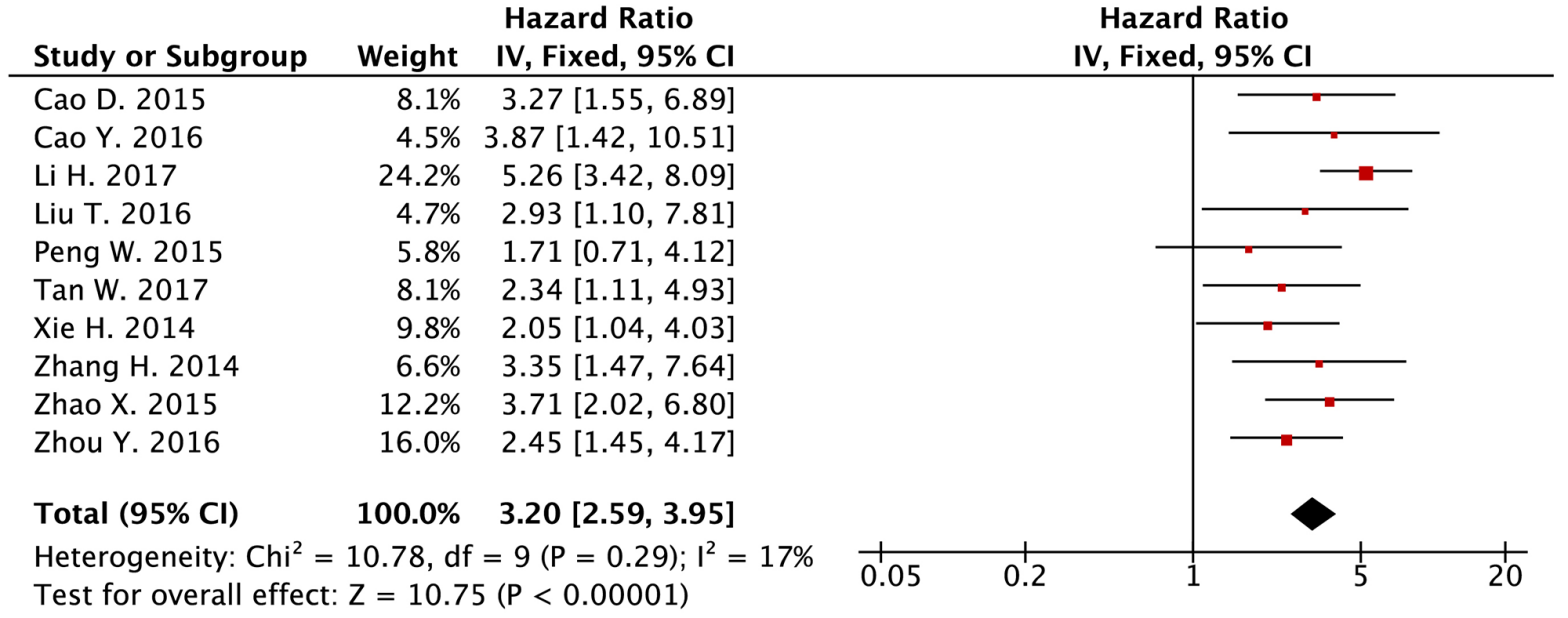
Forest plot of the association between lncRNA *SPRY4-IT1* expression level and overall survival in cancer patients from 10 studies (study for NSCLC was excluded)

**Figure 4:**
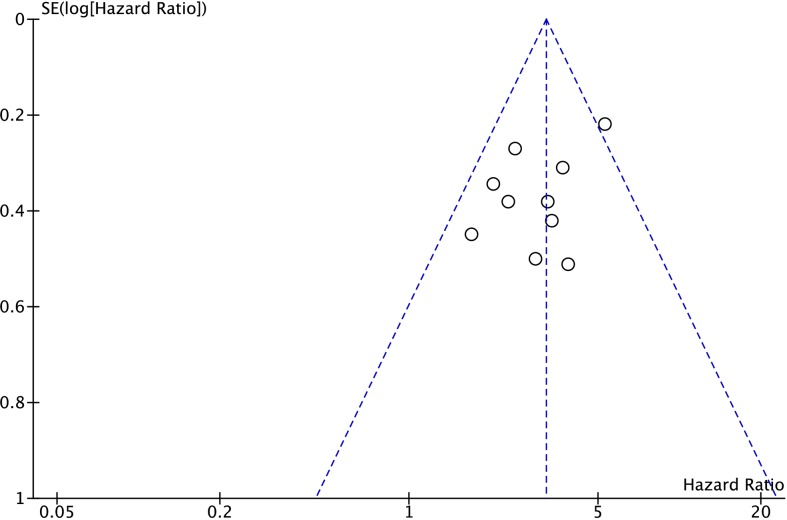
Funnel plot for assessing publication bias of the association between lncRNA *SPRY4-IT1* expression level and overall survival in cancer patients from 10 studies (study for NSCLC was excluded)

Eleven studies were included for the analysis of association between *SPRY4-IT1* expression level and OS in cancer patients. In the meta-analysis, random-effects model was applied to estimate the pooled hazard ratio (HR) and the respective 95% confident interval (CI), as heterogeneity exists among the 11 studies. As shown in Figure 2, the HR of the high *SPRY4-IT1* expression level group versus the low *SPRY4-IT1* expression level group was 2.45 (95% CI: 1.50-3.99) (Figure 2). After carefully reviewing the studies, we found that the study from Sun et al., 2014 showed a decrease of *SPRY4-IT1* in NSCLC tissues when compared to normal tissues, and down-regulation of *SPRY4-IT1* predicted shorter OS in patients with NSCLC. On the other hand, *SPRY4-IT1* was found to be up-regulated in the tissues from other studies, and up-regulation of *SPRY4-IT1* was positively correlated with shorter OS in these patients. In this regard, we excluded the study from Sun et al., 2014 [16], and the fixed-effects model was applied, as there was no heterogeneity in the analysis results (Figure 3). The HR of high *SPRY4-IT1* expression level versus the low *SPRY4-IT1* expression level group was 3.20 (95% CI: 2.59-3.95). The funnel plot analysis results showed that there was no obvious publication bias among these selected studies (Figure 4). Therefore, the study from Sun et al., 2014 was excluded in the following analysis.

**Figure 5:**
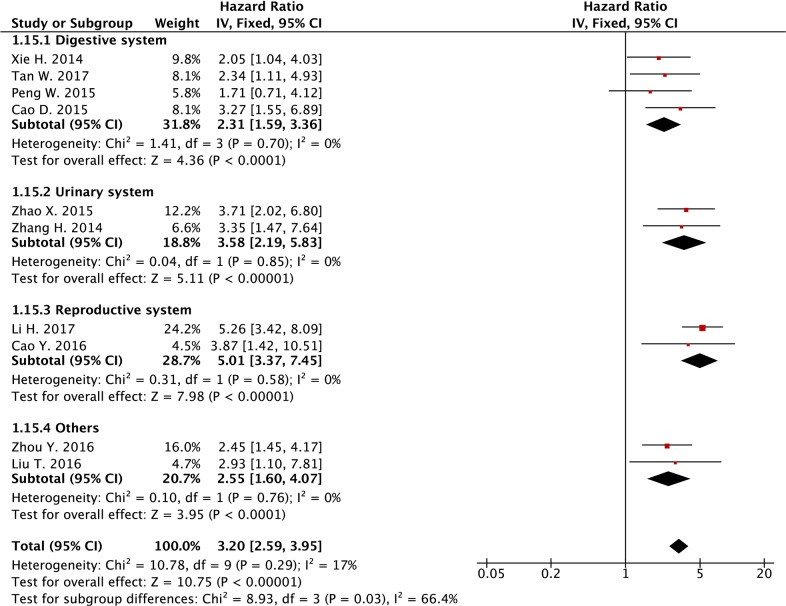
Forest plot of subgroup analysis (cancer type) for the association between lncRNA *SPRY4-IT1* expression level and overall survival in cancer patients

As shown in Supplementary Figure 1, there were nine types of cancer (bladder cancer, cervical cancer, colorectal cancer, esophageal squamous cell carcinoma (ESCC), gastric cancer, glioma, melanoma, ovarian cancer and renal cell carcinoma (RCC)) were included in the meta-analysis. We further classified these cancers into four subgroups (digestive system cancers, urinary system cancers, reproductive and other types of cancers), and the meta-analysis showed that the HR of the high *SPRY4-IT1* expression level group versus the low *SPRY4-IT1* expression level group in digest system cancers, urinary system cancer, reproductive system cancer and other types of cancer were 2.31 (95% CI: 1.59-3.36), 3.58 (95% CI: 2.19-5.83),), 5.01 (95% CI: 3.37-7.45), and 2.55 (95% CI:1.60-4.07), respectively (Table 2 and Figure 5), and there was no heterogeneity among studies from different subgroups (Table 2 and Figure 5). These results suggest that increased *SPRY4-IT1* expression level was associated with poor OS.

**Table 2:** Meta-analysis results of the association between the lncRNA *SPRY4-IT1* expression level and OS in cancer patients

Categories	Studies (n)	Number of patients	Fixed -effects model		Heterogeneity	
			HR(95% CI) for OS	*P*-value	I^2^ (%)	P_h_
[1] OS	10	1148	3.20 (2.59-3.95)	<0.001	17%	0.29
[2] Cancer type						
1) Digestive system	4	467	2.31 (1.59-3.36)	<0.001	0	0.70
2) Urinary system	2	166	3.58 (2.19-5.83)	<0.001	0	0.85
3) Reproductive system	2	234	5.01 (3.37-7.45)	<0.001	0	0.58
4) Others	2	281	2.55 (1.60-4.07)	<0.001	0	0.76
[3] Cut-off values						
Median	4	554	3.18 (2.40-4.23)	<0.001	69	0.02
Mean	3	282	3.15 (2.09-4.74)	<0.001	0	0.63
Others	3	312	3.32 (1.99-5.52)	<0.001	0	0.93
[4] Sample sizes						
≥ 100	5	688	3.03 (1.95-4.72)	<0.001	54	0.07
< 100	5	460	3.01 (2.17-4.19)	<0.001	0	0.77
[5] Year of survival						
3-year survival	1	84	3.27 (1.55-6.89)	0.002	-	-
5-year survival	9	1064	3.27 (1.94-2.91)	<0.001	84	<0.001
[6] Plasma vs. tissue						
Plasma	1	70	2.93 (1.10-7.81)	0.03	-	-
Tissue	9	1078	2.41 (1.97-2.94)	<0.001	84	<0.001

OS = overall survival

**Figure 6:**
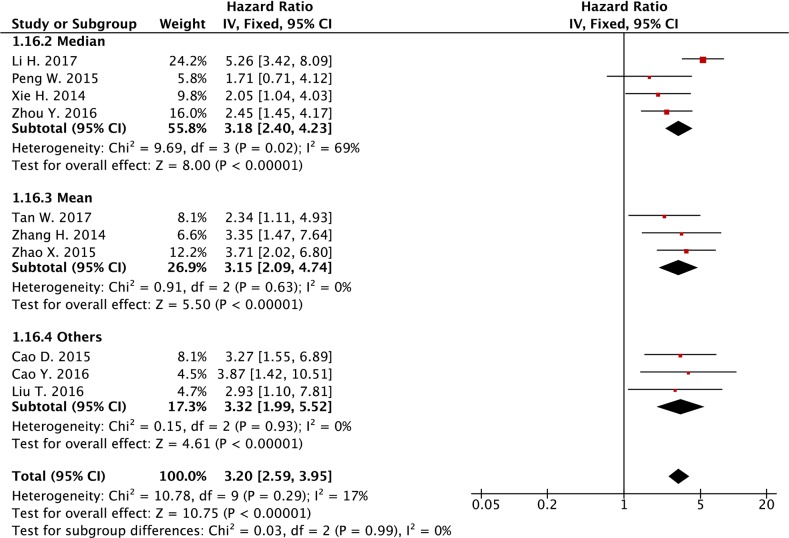
Forest plot of subgroup analysis (cut-off values) for the association between lncRNA *SPRY4-IT1* expression level and overall survival in cancer patients

**Figure 7:**
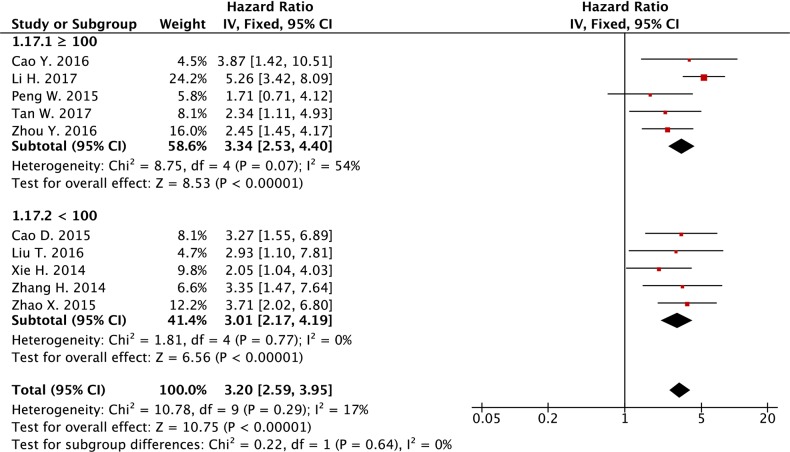
Forest plot of subgroup analysis (sample size) for the association between lncRNA *SPRY4-IT1* expression level and overall survival in cancer patients

In the further analysis, we also divided these studies into subgroups based on definition of cut-off values for *SPRY4-IT1* expression level, sample size of each study, 3 or 5 year overall survival, and plasma *SPRY4-IT1* versus tissue *SPRY4-IT1*, and we obtained similar results, in which the increased expression level was associated with poor overall survival in different subgroups divided based on above criteria (Table 2 and see forest plot of the analysis in Figure 6, Figure 7, Supplementary Figure 2 and 3).

### Sensitivity analysis

For the meta-analysis of the association between *SPRY4-IT1* expression level and OS, the sensitivity analysis was performed by removing each study in turn from the pooled analysis. This analysis aims to evaluate the impact of the removed study on the pooled HRs. In the present study, removing any of the included studies had no significant impact on the meta-analysis outcomes, which suggests the robustness of the results.

### Meta-analysis of the association between *SPRY4-IT1* expression level and disease-free survival (DFS)

**Figure 8:**
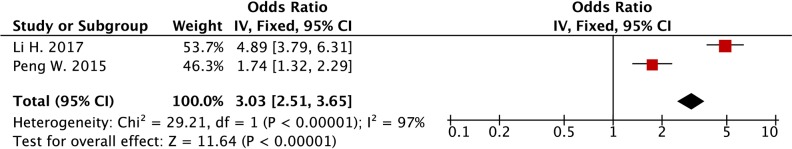
Forest plot of the association between lncRNA *SPRY4-IT1* expression level and disease-free survival in cancer patients

A total of 2 studies were included in the meta-analysis, and there are gastric cancer and ovarian cancer. The meta-analysis results showed that the HR of association between increased *SPRY4-IT1* expression level and DFS in these cancer patients was 3.03 (95% CI: 2.51-3.65), and I^2^= 97% and P_h_<0.001, suggesting that there is great heterogeneity existing between these studies (Figure 8). More data may be collected in the future to confirm the association between *SPRY4-IT1* expression level and DFS in cancer patients

### Meta-analysis of the association between *SPRY4-IT1* expression and clinical pathological parameters

We pooled all the clinicopathological data from these eligible studies to do further meta-analysis for the association between *SPRY4-IT1* expression level and clinicopathological characteristics. As shown in Table 3, the meta-analysis results showed that the *SPRY4-IT1* expression level was not correlated with the clinicopathological parameters including age (P = 0.37, Supplementary Figure 4), gender (P = 0.87, Supplementary Figure 5), tumor size (P = 0.47, Supplementary Figure 6) and invasion depth (P = 0.52, Supplementary Figure 7). However, the meta-analysis showed that the increased *SPRY4-IT1* expression level was significantly associated with distant metastasis (odds ratio (OR) = 1.96, 95% CI: 1.24-3.08, P = 0.004, Supplementary Figure 8), lymph node metastasis (OR = 3.96, 95% CI: 1.48-5.54, P<0.001, Supplementary Figure 9), advanced tumor/node/metastasis (TNM) stage (OR = 3.72, 95% CI = 2.91-4.76, P<0.001, Supplementary Figure 10), and poor tumor differentiation (OR = 1.86, 95% CI = 1.35-2.58, P<0.001, Supplementary Figure 11). Because of the insufficient data for other clinicopathological parameters (such as tumor location, family history of cancer, alcohol consumption), the relationship between increased *SPRY4-IT1* expression level and these clinicopathological parameters were not processed for the meta-analysis.

**Table 3:** Meta-analysis results for the association between the lncRNA *SPRY4-IT1* expression level and clinico-pathological parameters

Clinicopathological parameters	Studies (n)	Patients (n)	OR (95% CI)	*P*-value	Heterogeneity		
					I^2^ (%)	P_h_	Model
Age (≥ 55 vs. < 55 years)	12	1173	0.90 (0.70-1.14)	0.37	18	0.26	Fixed
Gender (Male vs. Female)	10	1067	0.98 (0.76-1.25)	0.87	0.51	0	Fixed
Tumor size (≥ 5 cm vs. <5 cm)	5	574	1.36 (0.59-3.15)	0.47	81	<0.001	Random
Invasion depth (T_3_-T_4_ vs. T_1_-T_2_)	3	341	1.85 (0.29-11.98)	0.52	90	<0.001	Random
Distant metastasis (Yes vs. No)	5	409	1.96 (1.24-3.08)	0.004	48	0.1	Fixed
Lymph node metastasis (Yes vs. No)	9	780	3.96 (1.48-5.54)	<0.001	18	0.28	Fixed
TNM stage (III-IV vs. I-II)	12	1065	3.72 (2.91- 4.76)	<0.001	23	0.22	Fixed
Tumor differentiation (Poor vs. Moderate/Well)	7	689	1.86 (1.35-2.58)	<0.001	35	0.16	Fixed

## DISCUSSION

The lncRNAs *SPRY4-IT1* is derived from an intron of the Sprouty 4 (*SPRY4*) gene [10]. *SPRY4-IT1* is located in the cytoplasm and is predicted to have several long hairpins in its secondary structure. Studies have suggested that *SPRY4-IT1* may act as molecular scaffolds for protein complexes that lack protein-protein interaction domains or can interact directly with microRNAs and prevent them from binding to mRNA, thus regulating protein synthesis [10]. In the aspect of cancer studies, *SPRY4-IT1* dysregulation was found to be closely associated with tumor development and also contributed to cell proliferation, cell apoptosis and cell invasion and cell migration in different types of cancers [10, 12–14, 17]. These findings may suggest the critical function of *SPRY4-IT1* in cancer progression and *SPRY4-IT1* may serve as a novel biomarker for early diagnosis and prognosis in cancer patients.

Several studies have elucidated the molecular mechanisms underlying *SPRY4-IT1* involved tumor development. SPRY4-TI1 was found to promote cell proliferation, migration and invasion via regulating epithelial–mesenchymal transition in various types of cancers including gastric cancer, colorectal cancer, ESCC, glioma and NSCLC [12, 16–19]. In addition, *SPRY4-IT1* also demonstrated the oncogenic role via targeting zinc finger protein 703 in breast cancer and ESCC [15, 20]. In the osteosarcoma, *SPRY4-IT1* can promote epithelial mesenchymal transition via interaction with *Snail* [21]. More importantly, the knock-down of *SPRY4-IT1* inhibited cell growth and cell differentiation, also induced apoptosis in melanoma [10]. In the aspect of the prognostic role of *SPRY4-IT1*, the increased expression of *SPRY4-IT1* was closely associated with poor prognosis in various types of cancers including bladder cancer, cervical cancer, colorectal cancer, ESCC, gastric cancer, glioma, melanoma, NSCLC and RCC [6, 11–13, 16, 18, 22–25]. Thus, the collective evidence may imply the oncogenic role of *SPRY4-IT1* in different types of cancers and targeting *SPRY4-IT1* may be beneficial for the treatment of human cancers.

In the present study, the meta-analysis results showed that increased *SPRY4-IT1* expression level was significantly associated with shorter OS, which suggests the prognostic role of *SPRY4-IT1* in predicting OS in cancer patients. Consistently, other lncRNAs such as *HOTAIR*, *H19* and *UCA1* were also found to predict the shorter OS in cancer patients [9, 26, 27]. In the future study, analysis of more than one lncRNAs may represent a better solution for predicting OS in cancer patients. Apart from the examining the role *SPRY4-IT1* in predicting OS, we also found that increased *SPRY4-IT1* expression level was also significantly correlated with shorter DFS in cancer patients. Similarly, the increased expression of the lncRNA *UCA1* also predicted the shorter DFS in patients with gastric cancer or HCC [28]. In addition, elevated lncRNA, *metastasis associated lung adenocarcinoma transcript 1* expression was also a significant predictor for DFS in patients with digestive system cancers [29]. For the lncRNA *HOTAIR*, its up-regulation also predicted the shorter DFS in cancer patients [30]. Therefore, these results may suggest that increased *SPRY4-IT1* expression level may predict the poor prognosis in various cancers.

Several lines of studies also showed the correlation between lncRNAs and clinicopathological parameters. Here, we showed that increased *SPRY4-IT1* expression was significantly associated with distant metastasis, lymph node metastasis, advanced TNM stage, and poor tumor differentiation. Indeed, *UCA1, PVT1 and H19* can serve as a molecular marker for lymph node metastasis in various cancers [9, 27, 31]. Liu et al., also found that the lncRNA, *low expression in tumor* was associated with lymph node metastasis and distant metastasis in human cancers [32]. All in all, our results may suggest that increased *SPRY4-IT1* may be associated with advanced features of cancer.

In the present study, there are still several limitations in the meta-analysis. For example, the total sample size was relatively small, and the patients included in the meta-analysis were all from one country. In addition, the cut-off definition for high *SPRY4-IT1* expression was not consistent among the included studies. Finally, publication bias may exist, despite the fact that no obvious publication bias was observed based on stable results revealed in sensitivity analysis as well as funnel plot analysis. All in all, larger-size, multi-center and higher-quality studies with unified criteria for defining *SPRY4-IT1* expression are essential to solidify the findings in this study. In the present study, only one study from Sun et al., 2014 [16] showed a decrease of *SPRY4-IT1* in cancer tissues and decreased expression of *SPRY4-IT1* was associated with poor clinical outcomes, which was contrast with other included studies. In addition, one study from Xie et al., 2015 [18] also showed a decrease of *SPRY4-IT1* in gastric cancer, which was not consistent with the study from Peng et al., 2015 [24], and after carefully reviewing the data from Xie et al., 2015 [18], we found that the description of results was not consistent with the figures (in Figure 1A from Xie et al., SPRY4-IT1 was up-regulated in gastric cancer tissue, while in the results section, the SPRY4-IT1 was described to be down-regulated in gastric cancer tissue), and this study was not included in the current meta-analysis.

In summary, the meta-analysis results suggest the prognostic role of *SPRY4-IT1* in human cancers, and increased *SPRY4-IT1* expression was closely associated with advanced features of human cancers except NSCLC. However, due to several limitations of the included studies, more high-quality studies may be required to further confirm our findings.

## MATERIALS AND METHODS

### Search strategy

Comprehensive literature search was performed in the following databases: Pubmed, EMBASE, CNKI, Web of Science and Google Scholar to retrieve potential eligible studies for meta-analysis and the cut-off date was defined as Feb, 2017. The keywords for the search in these databases included: “*SPRY4-IT1*”, “ *SPRY4 Intronic Transcript 1*”, “long non-coding RNA *SPRY4-IT1*”, “lncRNA *SPRY4-IT1*”, “cancer”, “tumor”, “carcinoma”, “neoplasm”, and other eligible studies were also manually retrieved from the relevant reference lists.

### Inclusion and exclusion criteria

Inclusion criteria for the eligible studies included: (a) associations of *SPRY4-IT1* expression levels with OS, DFS or clinicopathological features were described, (b) the role of *SPRY4-IT1* in human cancer development was examined, (c) patients were categorized into two groups based on high and low expression levels of *SPRY4-IT1*, (d) the expression levels of *SPRY4-IT1* in the cancer patients were determined by qRT-PCR. Exclusion criteria for the articles included: (a) studies without presenting data with relevant values, (b) duplicated publications, (c) letters, reviews, case reports and expert opinions.

### Data extraction and quality assessment

The data and information from all included eligible studies were independently assessed by two investigators (H.S. and N.S.). The following information were extracted from each eligible study: the name of first author, year of publication, cancer type, total number of patients from each eligible study, TNM stage, year of survival examined, criteria for defining high expression level of *SPRY4-IT1* and low expression level of *SPRY4-IT1*, method for detecting *SPRY4-IT1* expression, outcome measures, HR and its corresponding 95% CI, the clinicopathological parameters from each eligible study. In the eligible studies only reporting Kaplan-Meier curves, the software, Enguage Digitizer (Version 4.1) was used to extract the survival data. For the eligible studies that provided both the univariate and multivariate analysis, the multivariate values were chosen as the multivariate values had higher precision on interpreting confounding factors. In the situation of a disagreement, a consensus was reached by a third investigator (T.L.). The quality of all the included studies were assessed by The Newcastle-Ottawa Scale (NOS) method. The NOS scores ranged from 0 to 9, and a study with an NOS score more than 6 was regarded as high quality.

### Statistical analysis

The meta-analysis was performed with Stata SE12.0 and RevMan 5.3 software. The heterogeneity between studies was determined by the Chi square-based Q test and I^2^ statistics. *P*<0.05 for the Q test (P_h_) and I^2^>50% were considered to be significantly heterogeneous. The fixed effects model was applied in the studies with no obvious heterogeneity (P_h_>0.05, I^2^<50%); the random effects model was applied in the studies with obvious heterogeneity (P_h_≤0.05, I^2^≥50%). The sensitivity analysis was also carried out to assess the stability of the results. A *P* values less than 0.05 was considered to be statistically significant.

## SUPPLEMENTARY MATERIALS FIGURES


