# Supplementary material for: Prognostic role of the long non-coding RNA, SPRY4 Intronic Transcript 1, in patients with cancer: a meta-analysis
Source: Oncotarget. 2017 Mar 31;8(20):33713–24. doi: 10.18632/oncotarget.16735 (PMC5464905; doi:10.18632/oncotarget.16735)
Supplement: Supplementary file 1 [file oncotarget-08-33713-s001.pdf]

Prognostic role of the long non-coding RNA, *SPRY4 Intronic Transcript 1*, in patients with cancer: a meta-analysis

SUPPLEMENTARY FIGURES

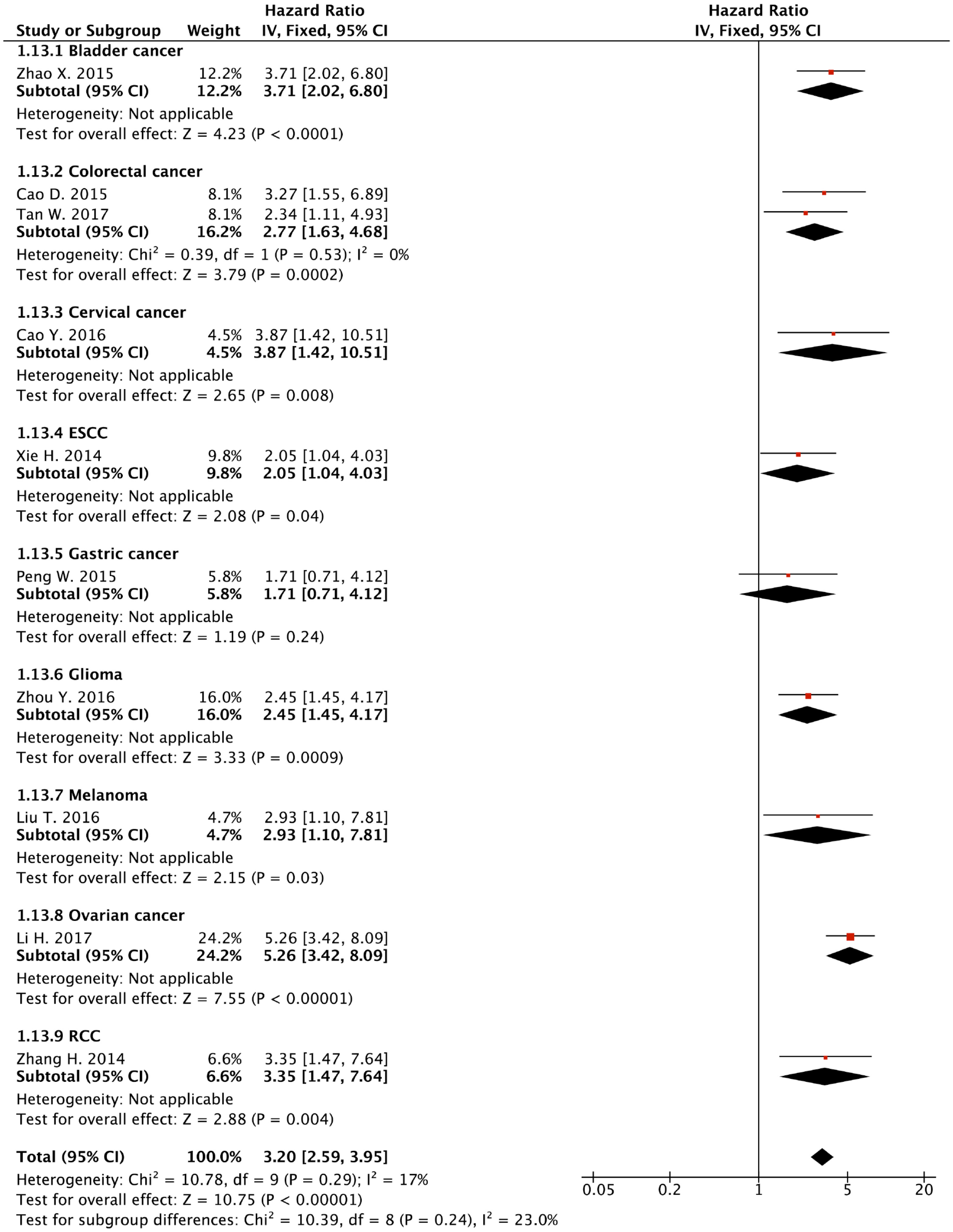

Supplementary Figure 1: Forest plot of different cancers for the association between lncRNA SPRY4-IT1 expression level and overall survival in cancer patients.

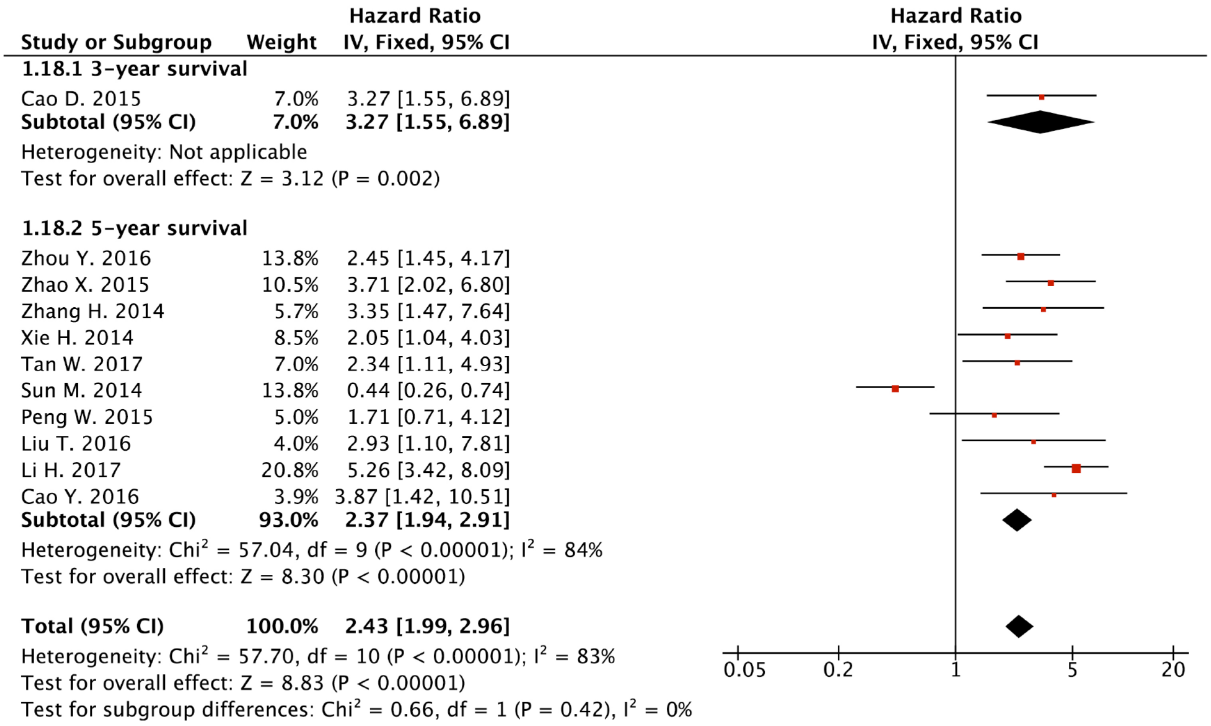

Supplementary Figure 2: Forest plot of subgroup analysis (follow-up duration) for the association between lncRNA SPRY4-IT1 expression level and overall survival in cancer patients.

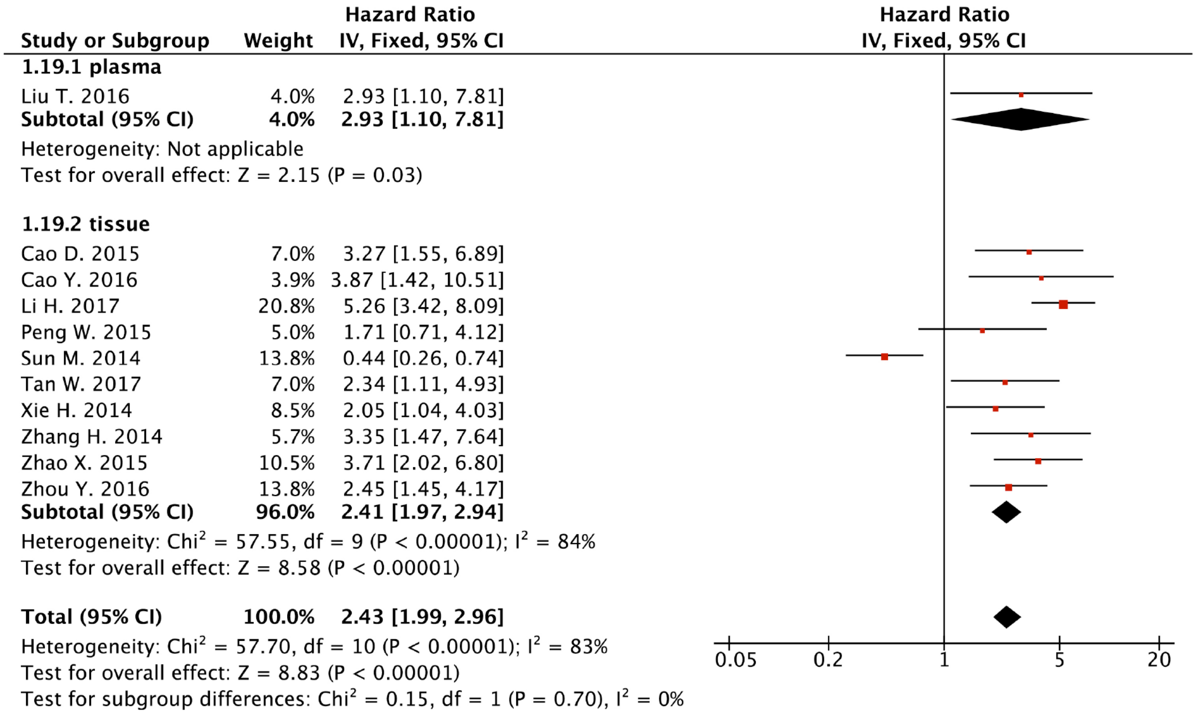

Supplementary Figure 3: Forest plot of subgroup analysis (plasma lncRNA vs. tissue lncRNA) for the association between lncRNA SPRY4-IT1 expression level and overall survival in cancer patients.

Study or Subgroup	Weight	Odds Ratio M-H, Fixed, 95% CI
Cao D. 2015	4.8%	1.56 [0.62, 3.96]
Cao Y. 2016	11.7%	0.48 [0.21, 1.06]
Li H. 2017	11.7%	0.77 [0.38, 1.57]
Liu D. 2017	2.0%	1.68 [0.42, 6.66]
Liu T. 2016	3.8%	2.00 [0.75, 5.33]
Peng W. 2015	15.0%	0.91 [0.50, 1.66]
Shi Y. 2016	4.8%	0.47 [0.13, 1.68]
Tan W. 2017	12.1%	0.48 [0.22, 1.06]
Xie H. 2014	9.3%	0.61 [0.26, 1.43]
Zhang H. 2014	7.5%	1.20 [0.54, 2.67]
Zhao X. 2015	5.7%	0.88 [0.33, 2.34]
Zhou Y. 2016	11.7%	1.32 [0.71, 2.46]
**Total (95% CI)**	**100.0%**	**0.90 [0.71, 1.14]**
Total events		

Heterogeneity: $Chi^2 = 13.47$, df = 11 (P = 0.26); $I^2 = 18\%$
Test for overall effect: Z = 0.90 (P = 0.37)

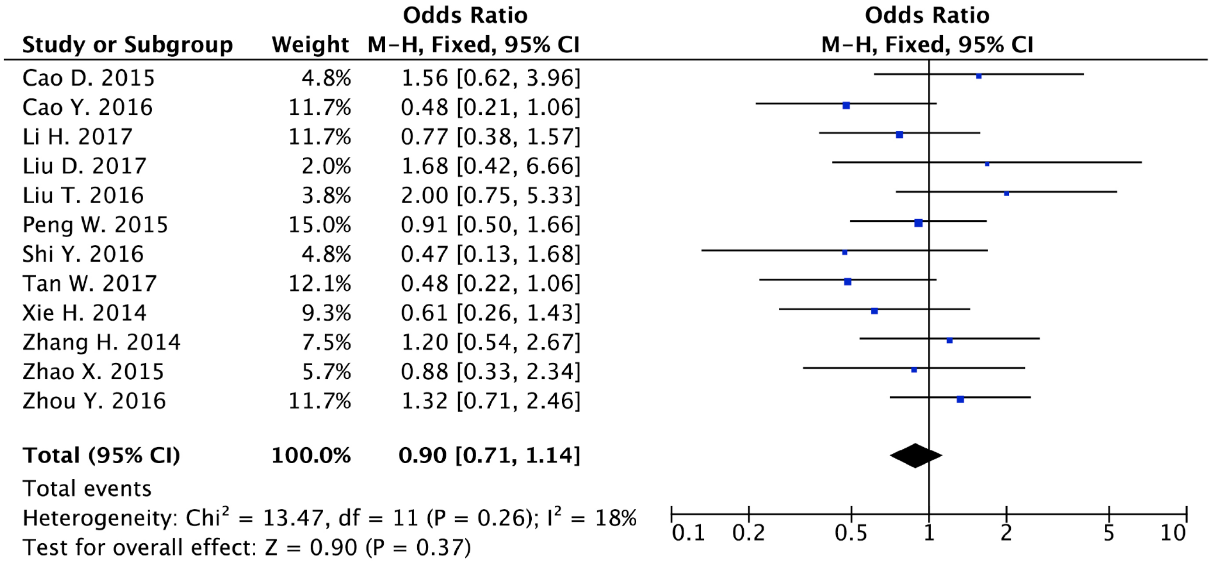

Supplementary Figure 4: Forest plot of the association between lncRNA SPRY4-IT1 expression level and age in cancer patients.

Study or Subgroup	Weight	Odds Ratio M-H, Fixed, 95% CI
Cao D. 2015	7.0%	1.29 [0.54, 3.08]
Liu D. 2017	6.1%	0.48 [0.15, 1.58]
Liu T. 2016	5.2%	1.48 [0.55, 3.96]
Peng W. 2015	15.5%	1.16 [0.63, 2.13]
Sun M. 2014	13.8%	0.68 [0.33, 1.41]
Tan W. 2017	15.4%	0.53 [0.26, 1.12]
Xie H. 2014	6.9%	1.57 [0.68, 3.65]
Zhang H. 2014	9.9%	0.94 [0.43, 2.10]
Zhao X. 2015	7.2%	0.83 [0.32, 2.18]
Zhou Y. 2016	13.1%	1.26 [0.66, 2.40]
**Total (95% CI)**	**100.0%**	**0.98 [0.76, 1.25]**
Total events		

Heterogeneity: $Chi^2 = 8.25$, df = 9 (P = 0.51); $I^2 = 0\%$
Test for overall effect: Z = 0.17 (P = 0.87)

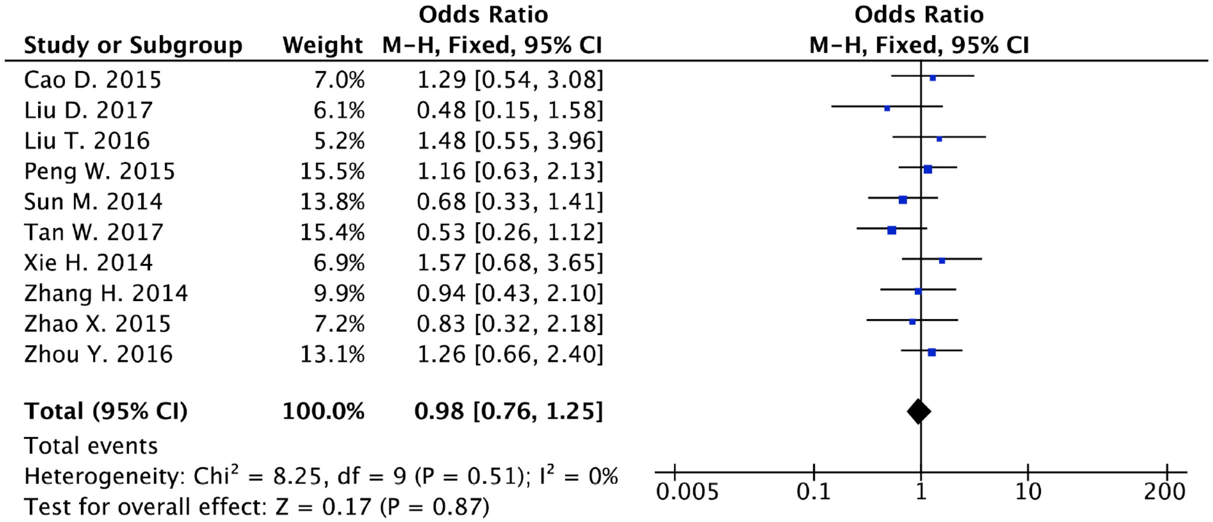

Supplementary Figure 5: Forest plot of the association between lncRNA SPRY4-IT1 expression level and gender in cancer patients.

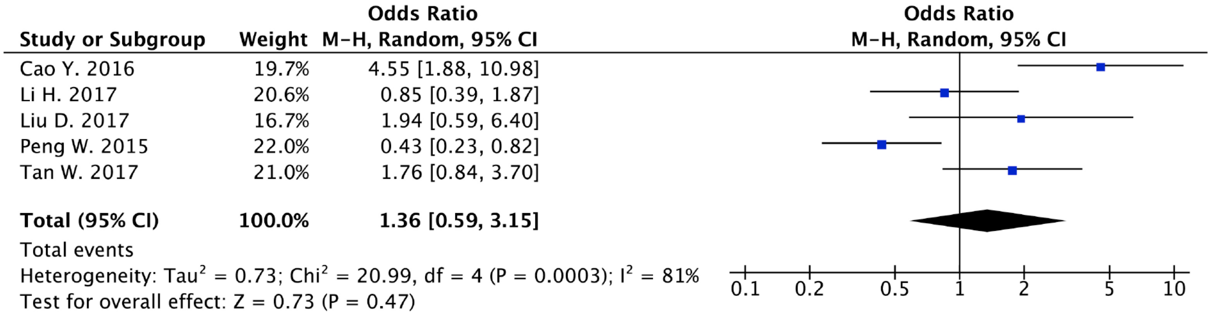

Supplementary Figure 6: Forest plot of the association between lncRNA SPRY4-IT1 expression level and tumor size in cancer patients.

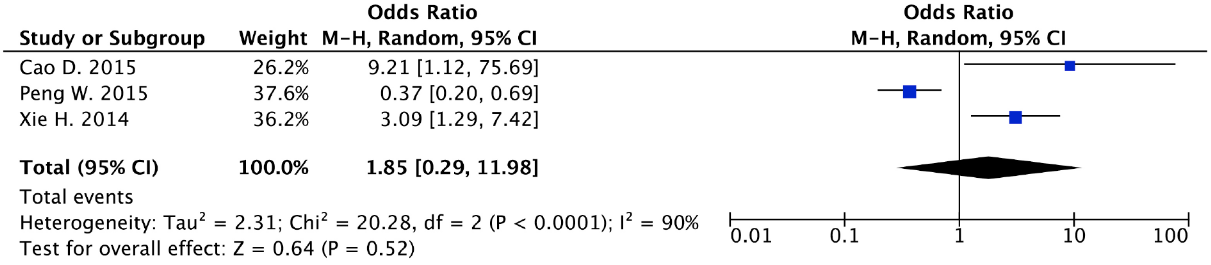

Supplementary Figure 7: Forest plot of the association between lncRNA SPRY4-IT1 expression level and invasion depth in cancer patients.

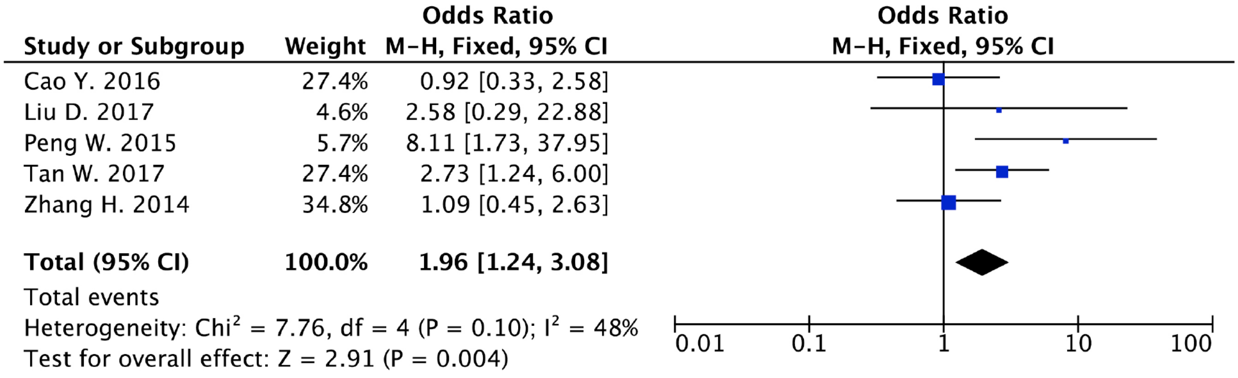

Supplementary Figure 8: Forest plot of the association between lncRNA SPRY4-IT1 expression level and distant metastasis in cancer patients.

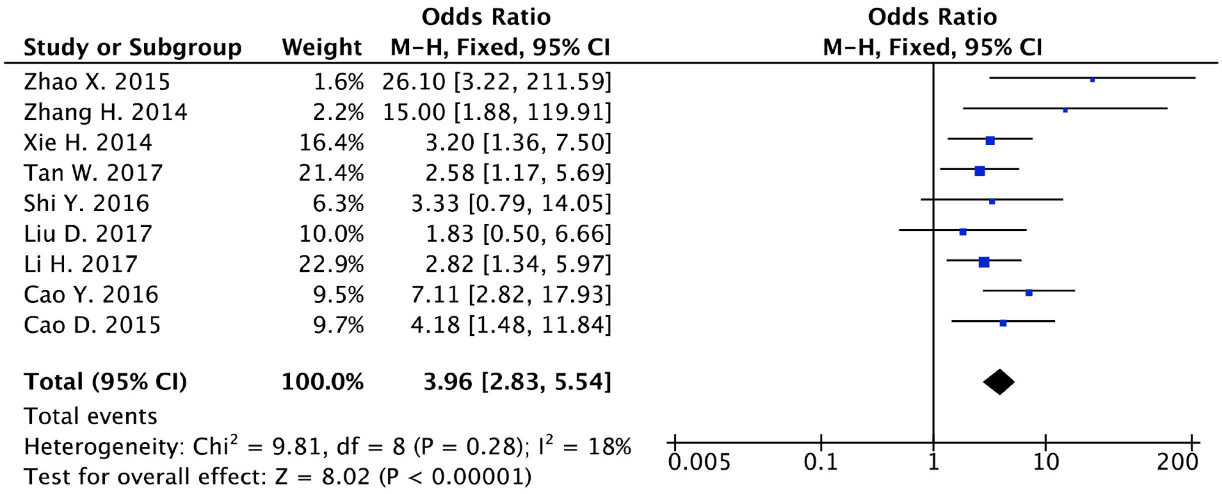

Supplementary Figure 9: Forest plot of the association between lncRNA SPRY4-IT1 expression level and lymph node metastasis in cancer patients.

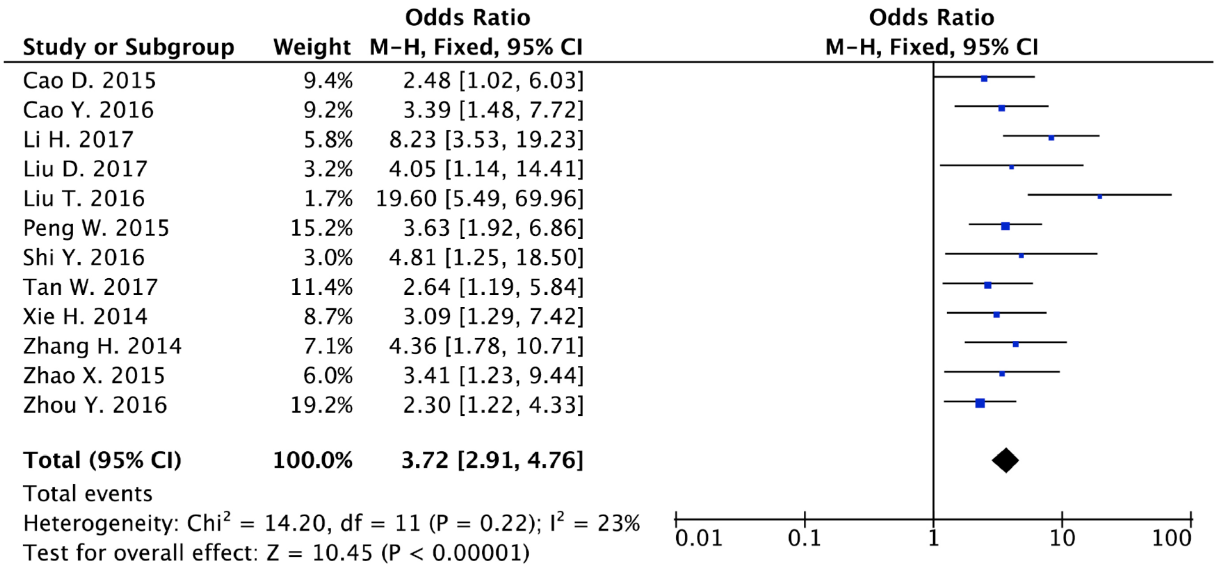

Supplementary Figure 10: Forest plot of the association between lncRNA SPRY4-IT1 expression level and TNM stage in cancer patients.

Study or Subgroup	Weight	Odds Ratio M–H, Fixed, 95% CI
Cao D. 2015	7.2%	2.33 [0.75, 7.30]
Cao Y. 2016	10.5%	2.58 [1.00, 6.63]
Peng W. 2015	33.1%	1.46 [0.80, 2.65]
Shi Y. 2016	6.2%	2.01 [0.57, 7.07]
Tan W. 2017	27.5%	0.93 [0.45, 1.95]
Xie H. 2014	9.8%	2.53 [0.96, 6.71]
Zhang H. 2014	5.7%	5.54 [1.93, 15.84]
**Total (95% CI)**	**100.0%**	**1.86 [1.35, 2.58]**
Total events		

Heterogeneity: $Chi^2 = 9.17$, df = 6 (P = 0.16); $I^2 = 35\%$
Test for overall effect: Z = 3.75 (P = 0.0002)

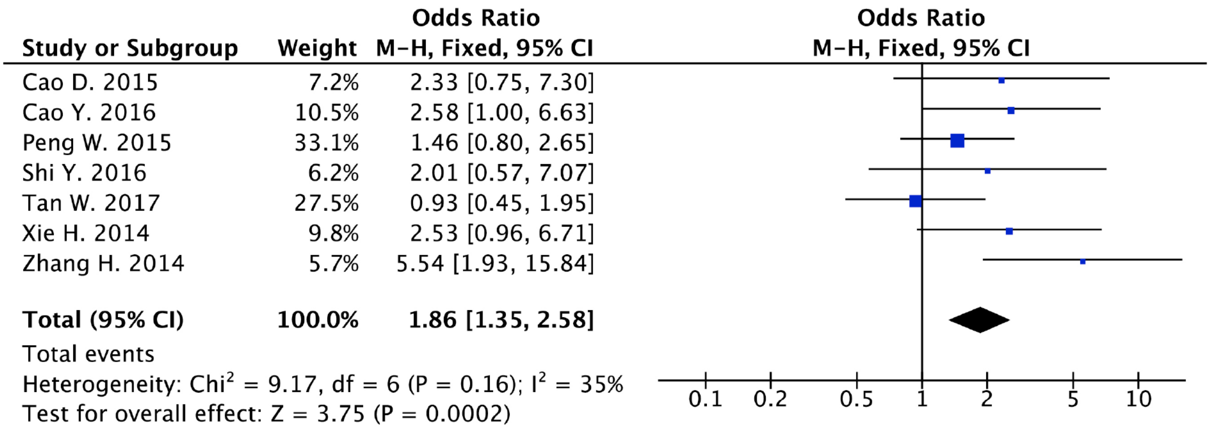

Supplementary Figure 11: Forest plot of the association between lncRNA SPRY4-IT1 expression level and tumor differentiation in cancer patients.
